# E-pharmacies in India: Can they improve the pharmaceutical service delivery?

**DOI:** 10.7189/jogh.10.010302

**Published:** 2019-06

**Authors:** Gautam Satheesh, Sandra Puthean, Vaibhav Chaudhary

**Affiliations:** 1 Department of Pharmacy Practice, National College of Pharmacy, Kozhikode, Kerala, India; 2 Alliance School of Law, Alliance University, Bangalore, Karnataka, India

Of late, Indian e-pharmacies, despite their emerging popularity and growing customer base, have suffered at the hands of the Indian judiciary. Although e-pharmacy constitutes only 3% of pharmaceutical sales in India, it has been subject to hostility from retail pharmacists’ associations such as All India Organization of Chemists and Druggists (AIOCD). In an attempt to formalize the online sale of drugs, the Union Health Ministry has recently laid down certain guidelines that require e-pharmacies to be registered with Central Drugs Standard Control Organization (CDSCO), the chief licensing and regulatory authority for pharmaceutical sales in India [1]. This move was met by strong retaliation from AIOCD, which even threatened to boycott the medicines marketed by those pharma-companies that invest in e-pharmacy [2].

Medicine costs, which account for about 70% of all health care costs in India, rarely get reimbursed because of very poor insurance coverage. Although health care initiatives by the Indian government (eg, *Jan Aushadhi* scheme) have improved affordability, the availability of medicines remains suboptimal in the public sector – forcing the majority to depend on the more expensive private sector. To solve this public health crisis, India needs a far more efficient pharmacy sector [3,4]. The rising chronic disease burden in urban India is also likely to expand the online pharmacy market, outdoing the retail counterparts. The heavy price concessions and a wide variety of brands offered by e-pharmacies had already begun to emerge as a potential solution to this crisis when the Delhi High Court banned online sale of medicines across the country in December 2018. The Indian judiciary, in good faith, opted to ensure safe pharmacy practice by acknowledging the possible risk of harmful self-medication and the sale of unregulated medicines through online platforms. This precautionary step against ever-expanding e-pharmacy was initiated after two writ petitions were filed [5,6]. The most recent regulatory development in the e-pharmacy sector only prioritizes the need for a valid prescription, while some of the major concerns remain unresolved.

With e-commerce blooming in India, the popularity of e-pharmacy too is on the rise, despite poorly defined regulations and unfavorable conditions for its growth. The sale of medicines in India is governed by the Drug & Cosmetics Act (1940) and Pharmacy Act (1948) – both of which were passed decades before the advent of the internet. Indian e-pharmacies, however, have been constantly on the radar of Drugs Controller General of India (DCGI) since 2016. This coincides with the USA’s initiatives to curb e-pharmacies by restricting online sale permits only to those sites that are National Association of Boards of Pharmacy (NABP) certified and also accredited though the Verified Internet Pharmacy Practice Sites (VIPPS) program [7]. However, the US laws have incorporated stricter rules than India to regulate the sale of pharmaceuticals online – for example, blocking money transactions through credit cards if the e-pharmacy is not NABP/VIPPS-certified.

**Figure:**
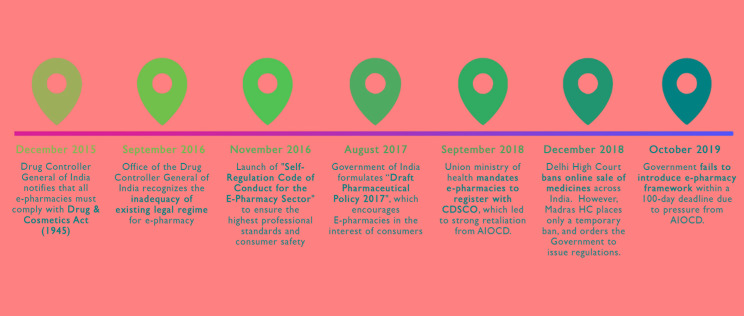
Photo: The ongoing predicament of e-pharmacies in India.

Most retail pharmacists challenge the concept of e-pharmacy by raising questions of safety, lack of dosage instructions, and potentially unregulated sale of prescription drugs. Yet, the retail pharmaceutical sector in India has inherent problems. Fundamental duties such as mandatory patient counseling have been ignored. A major problem is the authenticity of the pharmacist. While the law only states that a pharmacy should have a registered pharmacist, this does not stop the pharmacists’ relative or friend from providing a helping hand in the store. This augments the existing problem of many drugs of Schedule H, H1 and even X, being allegedly sold over the counter without a prescription. Another important advantage of e-pharmacies over retail pharmacies is how the former can indirectly improve medication adherence – especially among elderly patients with chronic diseases or disabilities – with timely door delivery of medicines.

Only a very small proportion of Indian retail pharmacists engage in patient counselling activities owing to various “counselling barriers”, ranging from tremendous workload to sheer inexperience. On the other hand, many Indian e-pharmacies (such as *1mg.com*) provide more extensive drug information online than what is available as “instructions on the box”. Patient counselling by pharmacists should be a fundamental requirement when it comes to the sale of medicines. Nevertheless, this is ignored by both policy makers and the judiciary. The caveat which pre-empts all discourse around this subject is that there is no mechanism in place to enforce mandatory patient counselling. Thus, the major challenge in the e-pharmacy sector posed by lack of drug information, requires a tailored solution, as opposed to the outright ban on e-pharmacies.

## CONCLUSIONS

By weighing the life-threatening risks of self-medication and dosing errors against the benefits of e-pharmacy, we propose – as a matter of public interest – that on-call pharmacists be made available to ensure validity of prescriptions as well as counsel e-pharmacy customers during drug purchase. This precondition for obtaining an e-pharmacy license will help eliminate significant concerns associated with online pharmacies. Moreover, if India succeeds in introducing a professionally rigorous system such as the NABP/VPPS, which can effectively monitor over 250 e-pharmacies that have been introduced recently, the overall health care expenditure can be brought down substantially [8]. An abundance of literature on the internet portrays e-pharmacies as a digital danger, and contributes to its longstanding negative perceptions. Considering India’s growing burden of communicable and non-communicable diseases, we aim to establish that e-pharmacies can help improve India’s unresolved medication access crisis. However, further research is warranted to confirm this and develop evidence-based policies for e-pharmacies.
